# Direct Reciprocity in Spatial Populations Enhances *R*-Reciprocity As Well As *ST*-Reciprocity

**DOI:** 10.1371/journal.pone.0071961

**Published:** 2013-08-07

**Authors:** Kohei Miyaji, Jun Tanimoto, Zhen Wang, Aya Hagishima, Naoki Ikegaya

**Affiliations:** 1 Interdisciplinary Graduate School of Engineering Sciences, Kyushu University, Kasuga-koen, Kasuga-shi, Fukuoka, Japan; 2 Department of Physics, Hong Kong Baptist University, Kowloon Tong, Hong Kong; 3 Center for Nonlinear Studies and the Beijing-Hong Kong-Singapore Joint Center for Nonlinear and Complex Systems (Hong Kong), Hong Kong Baptist University, Kowloon Tong, Hong Kong; University of Maribor, Slovenia

## Abstract

As is well-known, spatial reciprocity plays an important role in facilitating the emergence of cooperative traits, and the effect of direct reciprocity is also obvious for explaining the cooperation dynamics. However, how the combination of these two scenarios influences cooperation is still unclear. In the present work, we study the evolution of cooperation in 2×2 games via considering both spatial structured populations and direct reciprocity driven by the strategy with 1-memory length. Our results show that cooperation can be significantly facilitated on the whole parameter plane. For prisoner's dilemma game, cooperation dominates the system even at strong dilemma, where maximal social payoff is still realized. In this sense, R-reciprocity forms and it is robust to the extremely strong dilemma. Interestingly, when turning to chicken game, we find that *ST*-reciprocity is also guaranteed, through which social average payoff and cooperation is greatly enhanced. This reciprocity mechanism is supported by mean-field analysis and different interaction topologies. Thus, our study indicates that direct reciprocity in structured populations can be regarded as a more powerful factor for the sustainability of cooperation.

## Introduction

One major question in evolutionary biology and social science is to understand the emergence of cooperative traits and their sustenance under the pressure of free-rider. To explain the ubiquitous cooperation, a theoretical framework that has shed some light on this long-standing problem is the evolutionary game theory [1]–[3]. In particular, a simple, paradigmatic model, prisoner's dilemma game (PD), where two individuals simultaneously decide to adopt one of two strategies: cooperation (C) and defection (D), has attracted tremendous attention from both theoretical and experimental studies [4], [5]. When populations play the prisoner's dilemma game in the well-mixed case, this setup does not support the organization of cooperative dynamics. Over the past decides, a great number of scenarios have been identified that can offset an unfavorable outcome of social dilemmas and lead to the evolution of cooperation [6]–[10]. Whereas, Nowak attributed all these to five scenarios: kin selection, direct reciprocity, indirect reciprocity, network reciprocity, and group selection [11], which, comparing with the so-called well-mixed situation, can be somewhat related to the reduction of an opposing player's anonymity.

Among the five scenarios, network reciprocity, where players are arranged on the spatially structured topology and interact only with their direct neighbors, has attracted the greatest interest [12], because cooperators can survive by means of forming compact clusters which minimize the exploitation from defectors and protect those cooperators that are located in the interior of such clusters. Along this seminal idea, the role of spatial structure, and its various underlying variance in evolutionary games, have been intensively explored (see [13], [14] for a recent review). In addition, scientists also find that the strategy updating rule and dynamics on spatial topology also take a significant impact on the evolution of cooperation [15]–[28]. Let us mention a couple of typical examples. In recent research works [29]–[33], where players were allowed to adjust their strategy based on diverse learning ability or aspiration to fittest opponent, the prevalence of cooperative behavior even under large temptation to defect was observed. In [34] it was reported that the replicator dynamics could lead to an outbreak of cooperation on complex network, even if the conditions did not necessarily favor the spreading of cooperators. It was promising, furthermore, strategy update rules as well as update dynamics were more influence on the evolution of cooperation than the network topology alone [35], [36]. In [37], [38], allowing weight into evaluation of individual fitness, cooperation was also largely enhanced.

Interestingly, except for the above studies mostly focusing on the prisoner's dilemma game (PD), other paradigmatic settings have also been explored on top of spatial topology [39]–[44]. Of particular renown are the investigations of chicken game (CH) (or snowdrift game (SD)) [45], where the best action for individual relies on the choice of your opponent: to defect (cooperate) if the other cooperates (defects). Such a case finally leads to the coexistence of cooperators and defectors, namely, the state of *ST*-reciprocity [46], which is preferable to maximize population payoff than *R*-reciprocity [47], [48], [49]. To explain the social cooperative behaviors in this game, many different proposals aimed at sustaining cooperation were suggested and investigated. Examples include continuous strategy [50] multi-person interaction [51], stochastic noise in the payoff [52], teaching activity [53], [54], mobility [55]–[57], memory [41] and fitness evaluation [58], to name but a few.

In spite of the relative body of work that has been accumulated in the past years, the study for supporting cooperation traits is usually separated with the framework of prisoner's dilemma game (PD) or chicken game (CH). The situation of resolving the social dilemma in both games remains less explored [59], because an effective approach in one game may not provide a way for cooperation to survive in other game. Moreover, in realistic society, the type of dilemma is variable and more complex, how to constitute a universal protocol facilitating cooperation becomes highly necessary and meaningful. Inspired by all these, in the present work, we introduce the mixed strategy with 1-memory length into the different spatial game classes to study the evolution of cooperation, where both network reciprocity and direct reciprocity induced by the memory are suggested. We explore whether cooperation is sustained, especially for prisoner's dilemma game (PD) and chicken game (CH). Our results show that cooperation is actually promoted under such a protocol. In the remainder of this paper we will first describe the considered evolutionary games, subsequently, we will present the main results, and finally we will summarize our conclusions.

## Model

**Figure 1:**
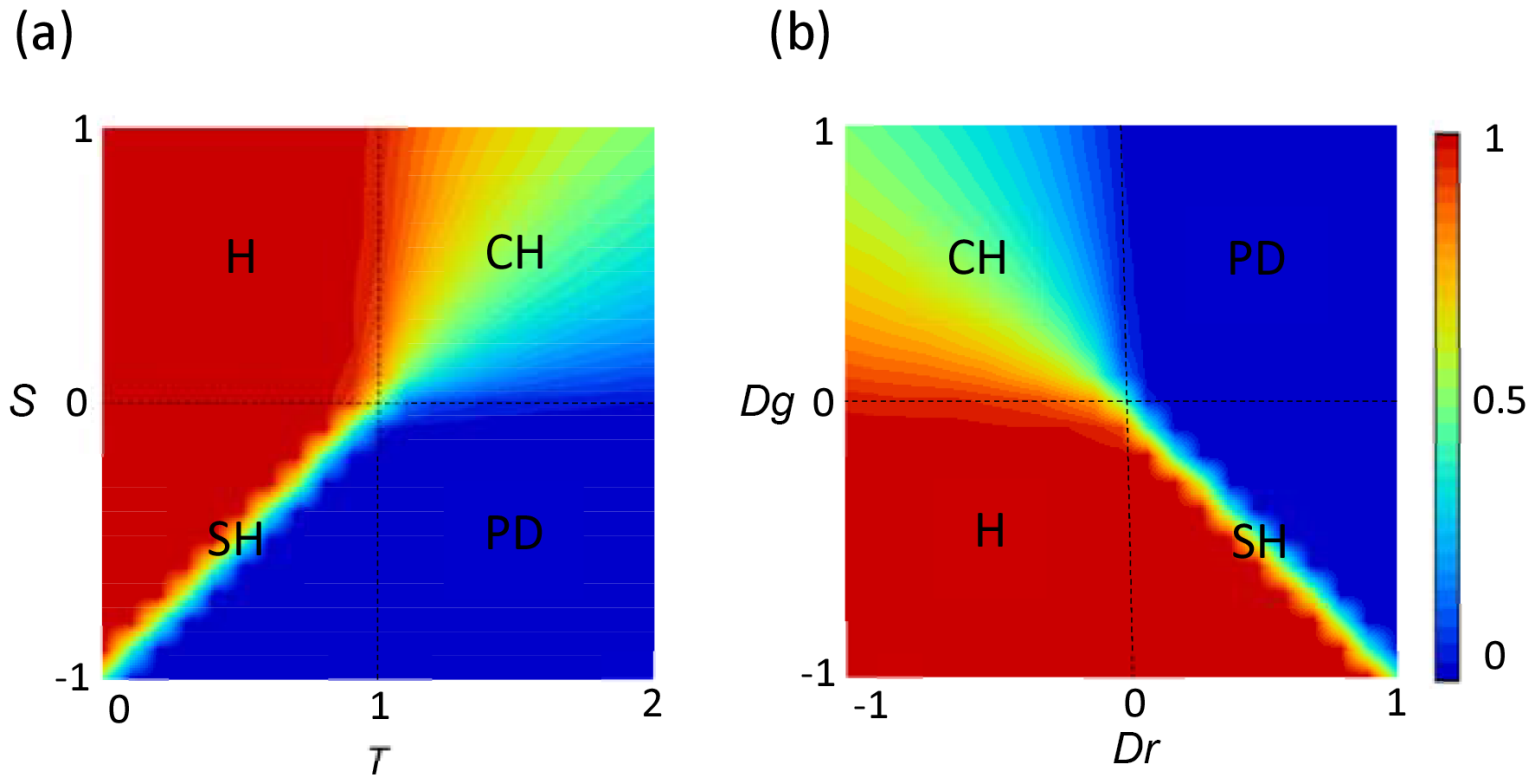
Schematic presentation for fraction of cooperators 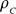 in well-mixed populations derived by replicator dynamics. (a) is illustrated by *T*-*S* parameter plane, and (b) is illustrated by *D_g_*-*D_r_* parameter plane, where PD, CH, SH and H denote prisoner's dilemma game, chicken game, stag-hunt game and harmony game, respectively.

We consider 2×2 game as the archetype. In order to depart from the traditional setup of spatial social dilemma games, we introduce strategy profile 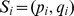
to each player, where 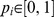
is the probability that player *i* will cooperate with player *j* if agent *j* was a cooperator in the last step, while 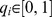
is the probability that player *i* will cooperate with player *j* when player *j* defected in the anterior step. Interestingly, 1-length memory is assumed for each player to store the opponent's previous strategy, and it can be, to some extent, considered as a type of mixed strategy game with 1-memory length. In a typical game, two players simultaneously decide whether they wish to cooperate or defect. If both cooperate (defect) they receive the reward *R* (the punishment *P*). If, however, one player chooses cooperation while the other defects, the latter gets the temptation *T* and the former is left with the sucker's payoff *S*. For simplicity, the standard scaled parameterization entails designating *R* = 1 and *P* = 0 as fixed, while the remaining two payoffs can be occupied −1≤*S*≤1 and 0≤*T*≤2. Thus, if *T*>*R*>*P*>*S* we have prisoner's dilemma game (PD), *T*>*R*>*S*>*P* yields chicken game (CH) (or snowdrift game (SD)), and *R*>*T*>*P*>*S* belongs to stag-hunt game (SH), as schematically presented in Fig. 1(a). Without loss of generality, the payoff parameterization can also been denoted by the stag-hunt-type dilemma *D_r_* = *P*−*S* and the chicken-type dilemma *D_g_* = *T*−*R* as follows [48], [60], 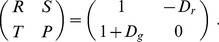
(1)


Correspondingly, we have the prisoner's dilemma game (PD) if 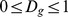
and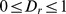, the chicken game (CH) if 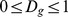
and 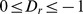, the stag-hunt game (SH) if 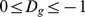
and 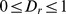
(see fig. 1(b)).

Throughout this work each player *i* is initially designated either as a cooperator (*C*) or defector (*D*) with equal probability, and is also assigned the parameter value *S* = (*p*, *q*) to the interval [0, 1]. This setting is performed uniformly irrespective of its initial strategy and remains unchanged during the simulations. As the interaction network, we use either the 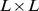
regular square lattice or random regular graph (RRG) constructed as described in [61]. At each Monte Carlo step (MCS), defined as the amount of time, on average, each player has a chance to update its strategy once. The updating procedure comprises the following elementary steps. First, a randomly chosen player *i* earns its payoff 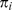
by playing the game with all its four neighbors. Then, we evaluate in the same way the payoffs of all the neighbors of player *i*. At last, player *i* adopts the strategy from the selected player *j* with the probability 
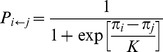
(2)where *K* denotes the amplitude of noise [62]. The effect of noise on the cooperation in the spatial game has been studied in detail in previous work [63]. Since this issue goes beyond the purpose of the present work, in all our following studies, we simply fix the value of *K* to be *K* = 0.5.

The results of Monte Carlo simulations presented below are obtained for lattices with 100^2^ individuals, and the average fraction of cooperators 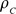, that is, the number of cooperators divided by 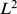, is determined by the average within the last 2000 steps out of the total 2×10^5^ MCS. Moreover, since the random distributions of *p* and *q* may introduce additional disturbances, the final results are averaged over up to 100 independent runs for each set of parameter values in order to assure suitable accuracy.

## Results and Discussion

**Figure 2:**
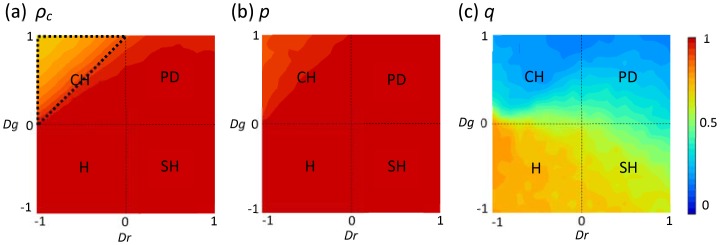
Color map depicting (a) fraction of cooperation 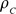, (b) *p*, and (c) *q* on the *D_g_*-*D_r_* parameter plane. Obviously, in the top left corner of CH where *D_g_>D_r_+1* (or *2R*<*S*+*T*) is satisfied (surrounded by black dotted line), incomplete cooperation is emerged (namely, the so-called *ST*-reciprocity [46]), which can lead to high payoff than the case of complete cooperation (the so-called *R*-reciprocity [47]).

We start by presenting the color map encoding the final fraction of cooperation 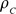, strategy profile *p* and *q* on the *D_g_*-*D_r_* parameter plane in Figure 2. It is obvious, compared with the solution of well-mixed population shown in Figure 1, cooperative behavior drastically enhances in our setting. Even under the case of strong dilemma *D_g_* = *D_r_* = 1 (PD region), where mutual defection dominates in the traditional scenario, almost complete cooperation can be observed. In this sense, the prosperity of cooperative behavior suggests the formation of *R*-reciprocity, where the best choice to maximize social profit is that all players become cooperators to obtain R in prisoner's dilemma game [46], [48], [49]. Moreover, it is interesting to focus on the strategy profile parameters. *p* reaches nearly 1 besides the top left corner of CH, while *q* differs according to the exposed dilemma strength and gradually gets close to 0 when the chicken-type dilemma *D_g_* exceeds 0. Based on these facts, the elucidation for the high level of cooperation is explicit that the defined strategies in our model can be regarded as the mixed strategies, which effectively help cooperators to weaken defector attacks. Naturally, such a feedback mechanism causes the preference of cooperation with a defector (*i.e.*, the value of *q*) fast decreasing. Thus, we argue when stochasticity is introduced in the decision making process, the evolution of cooperation thrives.

**Figure 3:**
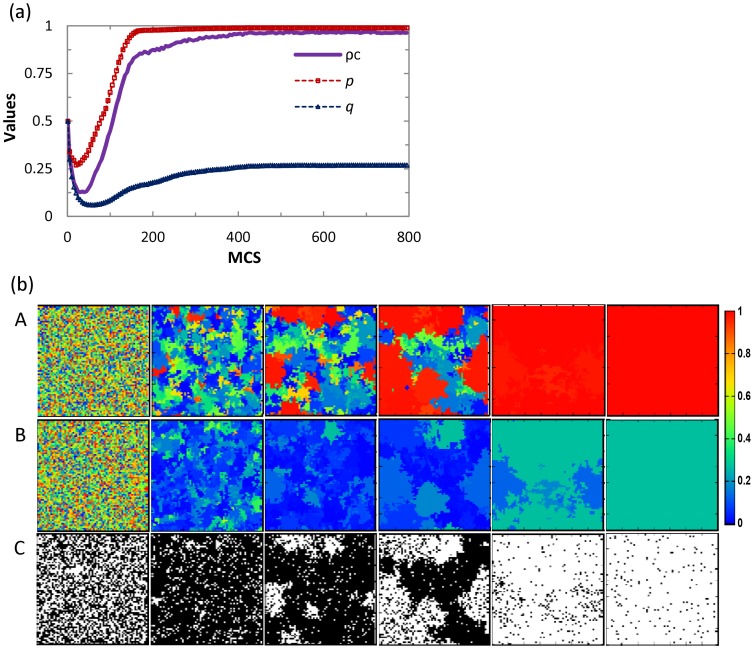
(a) Time courses for the evolution of cooperation and strategy profile *p, q* at *Dg* = *Dr* = 1. (b) The corresponding snapshots for the corresponding evolution course. (b)-A shows parameter *p*; (b)-B shows parameter *q* and the behaviors of cooperators (C, white) or defectors (D, black) are presented in the panel of (b)-C. For these snapshots, the time steps from left to right are 0, 20, 60, 100, 300 and 700, respectively.

In order to explain the promotive impact of mixed strategy (caused by memory) on the evolution of cooperation, we examine the evolution process of cooperation fraction 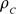, strategy profile *p* and *q*. Figure 3 features results obtained for *D_g_* = *D_r_* = 1, whereat the corresponding behavioral snapshots are shown as well (see Fig. 3(b)-C). Interestingly, as observed in the traditional version [29], [30], [62], [64], in the early stages of the evolutionary process, it appears as if defectors would actually fare better than cooperators. This is actually in agreement with what one would expect, given that defectors are, as individuals, more successful than cooperators and will thus be chosen more likely as potential strategy donors. At the same time, we can observe that the values of *p* and *q* decrease. However, the tide changes fast, as one can observe from the presented time series, the individuals with high *p* value start to form compact clusters (see the Fig. 3(b)-A), which, to large extent, helps more agents choose cooperation to resist the disadvantageous environment. Under the guidance of such a direct reciprocity proposal, the few remaining clusters of cooperators start recovering lost ground against expended defectors. More crucial is the fact that the clusters formed by these cooperators are impervious to defector attacks again, which can obtain sufficient attestation through the extremely low *q* value. In a sea of cooperators another cooperator rather than a defector always tries to penetrate into the clusters. Thus, we validate our argument that the feedback mechanism driven by direct reciprocity scenario halts and transfers the march of defectors to the undisputed decay. This newly identified mechanism eventually leads to the widespread cooperation that goes beyond what can be warranted by the spatial reciprocity alone [48], [49].

Next, it is interesting to focus on the evolution of cooperation in chicken game (CH). One notable character is that the complete cooperation phase (namely, 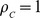) is still not observed in the upper half part of CH (surrounded by dotted line in Fig.2(a)) even if both memory and spatial topology are implemented. What happens in the game? Here, to obtain more payoff, *ST*-reciprocity becomes more meaningful than *R*-reciprocity when the condition *D_g_*>*D_r_*+1 (or *2R*<*S*+*T*) is satisfied. In what follows, we will systematically examine the validity of this claim.

To quantify the vantage of maintaining *ST*-reciprocity in chicken game (CH), we first calculate the expected payoff via the mean-field approximation. Assuming the cooperation fraction at equilibrium as 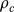, then the expected payoff for each individual 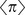 should be 
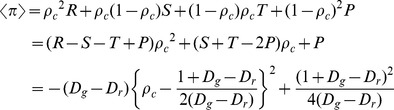
(3)


**Figure 4:**
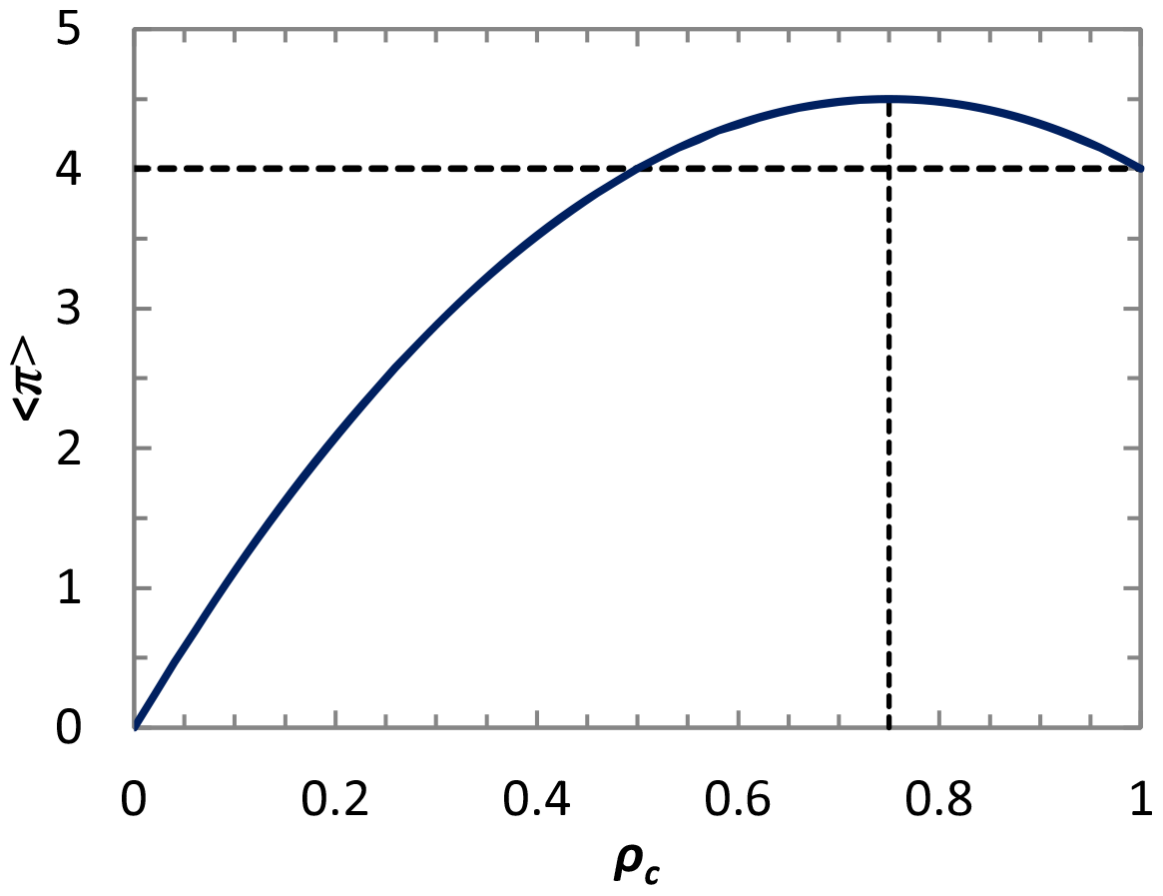
Expected payoff 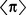 as a function for fraction of cooperation 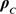 at *D_g_*  =  *−D_r_*  = 1, which is obtained by applying mean-field approximation approach. It is a quadratic function curve. Therefore, there is a cooperation fraction 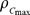
to guarantee the highest expected payoff 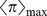. For example, in the Fig. 4, best social payoff 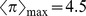 (exceeding 4R) is supported by 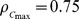 (indicated by dotted line.).

According to the above expression, we can further obtain the maximal payoff 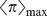 and the corresponding cooperation fraction 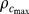
resulting in this maximal payoff 
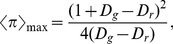
(4)and



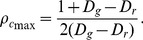
(5)
Figure 4 illustrates how the expected payoff 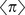
obtained by playing games with four neighbors varies as a function of cooperation fraction 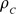
when assuming *D_g_* = *−D_r_* = 1. Obviously, 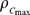
can maximize the average social payoff in the population, because the expected payoff is a quadratic function curve for the fraction of cooperation. We also confirm that when the spatial structure is introduced, the final distribution of agents' strategies is homogeneous at equilibrium (due to the fact that the continuous value is permitted as strategy profile). Thus, the discussion about mean-field approximation is still valid in spatial structure. Substituting *D_g_* = *−D_r_* = *x* into both Eqs. (4) and (5), we obtain the maximum payoff and the corresponding cooperation fraction as follows, 
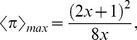
(6)

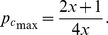
(7)


**Figure 5:**
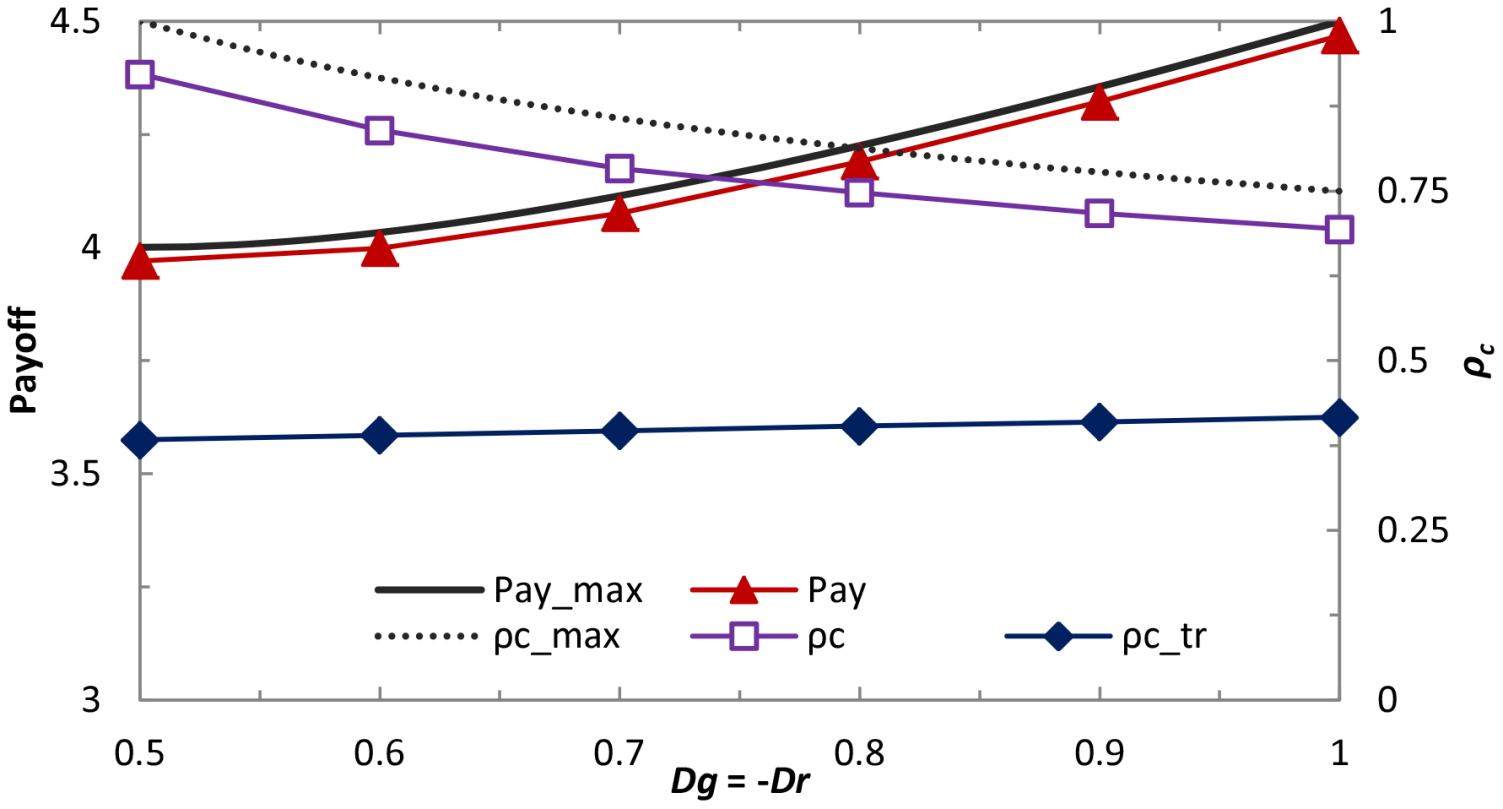
Comparison between simulation results and analytical solutions derived by mean-field approximation approach in chicken game (CH). Solid line (Pay_max) and dotted line (ρc_max) show maximum payoff and the corresponding cooperation fraction for analysis, respectively. Triangles (Pay) and squares (ρc) show simulation results for maximal payoff and the corresponding cooperation fraction. Diamonds (ρc_tr) show simulation results on square lattice when traditional discrete strategy setting is used.

We need to particularly note that these formulas are only valid for *D_g_*>*D_r_*+1 (or *2R*<*S*+*T*), because this limitation guarantees *ST*-reciprocity becoming more meaningful (to obtain higher payoff) than *R*-reciprocity. Figure 5 features the comparison between the theoretical analysis and the simulation result. It is evident that the average payoff under simulation is close to the theoretical maximum payoff. Moreover, because of the well-known claim that spatial topology may inhibit the evolution of cooperation in the chicken game (CH) (or snowdrift game (SD)) [45], it becomes of interest to explore the *ST*-reciprocity. We can observe, under the joint impact of spatial interaction topology and direct reciprocity driven by memory, that efficient *ST*-reciprocity (that exceeding *R*-reciprocity) can be maintained.

**Figure 6:**
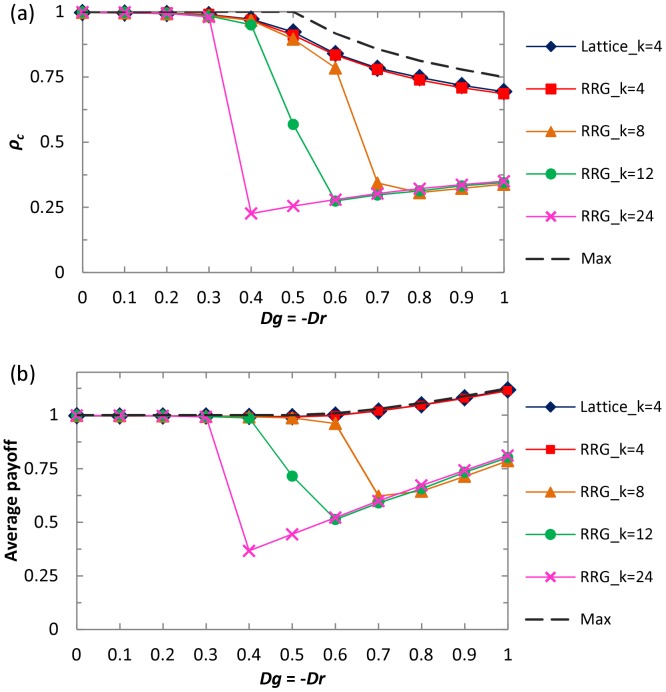
(a) fraction of cooperation 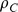
and (b) average payoff when random regular graph (RRG) is employed as the interaction network topology. For comparison, the theoretically predicted maximum payoff and its cooperation fraction (dashed line, denoted by “Max”) are depicted.

**Figure 7:**
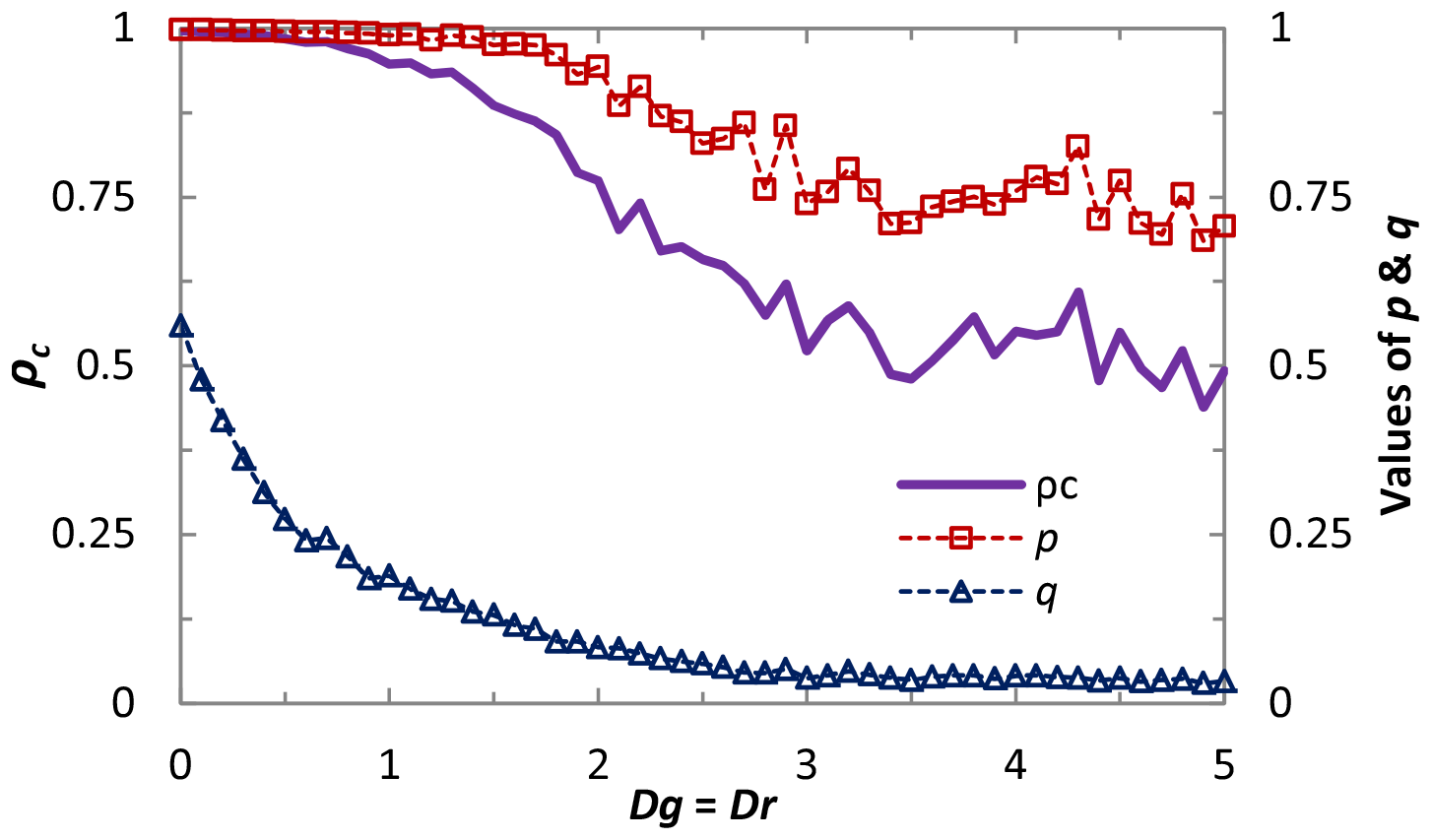
Fraction of cooperation 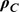 and strategy parameters, *p* and *q* in dependence on dilemma strength. Obviously, cooperative behavior can be remained even under extremely strong dilemma structure when both memory length and the network are assumed.

An important remaining question is to examine the universality of mixed strategy implemented by two parameters within different topology and neighborhoods. Results presented in Fig. 6 depict how cooperators and the average payoff fare on the random regular graph (RRG). Similarly as Fig. 2(a), it can be observed, when the condition *Dg*>*Dr*+1 is satisfied, that cooperators perform significantly better than the well-mixed case yet can not reach the complete dominance. While for the average payoff, strikingly, we can observe that it becomes more profitable than the state of full cooperation, which proves the existence of *ST*-reciprocity once again. This is in the qualitative agreement with the observations made on the square lattice, indicating that direct reciprocity in spatial populations is universally effective in promoting the evolution of cooperation and enhancing *ST*-reciprocity, irrespective of the underlying interaction networks. In addition, we can observe that, with the increment of neighborhood, cooperation fraction will decay and corresponding average payoff becomes lower, which is consistent with previous prediction of mean field approximation [65]. Lastly, it is instructive to explore how the cooperation evolves under extremely strong dilemma. Figure 7 shows the cooperation behaviors and strategy profile *p*, *q* as a function of *D_g_* value. Strikingly, full cooperation dominance state can be maintained even for *D_g_*>1, which further supports the fact that the newly introduced scenario about the reciprocity in spatial topology boosts the *R*-reciprocity and is generally valid for strong dilemma. When *D_g_* is sufficiently large (namely, *D_g_*>1.8), the cooperation level within the system starts to decline slowly, and *p* possess the similar tendency with the changing of *ρ_c_* (note that the downfall of *q* is particularly obvious in the weak dilemma region and its value approximates to 0 for extremely strong dilemma). Thus, direct reciprocity in spatial populations, i.e., the propensity of individual cooperation with the opponent according to previous performance, can be seen as a universally applicable promoter of cooperation for different dilemma games.

## Conclusion

We have presented a new framework of direct reciprocity on spatial populations in 2×2 games, where two strategy profile parameters *p* and *q* are taken into account. By means of extensive simulations, we have found, to maximize social efficiency, that agents alternatively change their strategies according to the difference of exposed dilemma structure, which is even effective under the strong dilemma structure. Compared with the case of spatial reciprocity alone [36], it is interesting that complete cooperative phase can be maintained till extremely strong dilemma structure in prisoner's dilemma game (PD). The elucidation for the promotion of cooperation can be attributed to a feedback mechanism: the survival cooperators not only induce a collective resistance against the invasion of defectors, but importantly accelerate the formation of extremely robust clusters of cooperators, where they are more likely to be regarded as the potential strategy donors and surrounded by more followers. In this sense, the area of *R*-reciprocity extensively increases (*i.e.*, players still choose mutual cooperation for obtaining *R* in strong dilemma). Moreover, another interesting finding is that although cooperation trait cannot reach the perfect state in the region *D_g_*>*D_r_*+1 (or *2R*<*S*+*T*) of chicken game (CH), *ST*-reciprocity can be guaranteed (i.e., alternatively obtaining *S* and *T* is more profitable than mutual cooperation), which is robust to the network topology. Through mean-field analysis, we have also proved that social average payoff has maximum value in this particular area. Therefore, the direct reciprocity in spatial populations can be regarded as a universally applicable promoter of cooperation irrespective of the evolutionary games. We hope that it will inspire future studies, especially in terms of the solution of some realistic social puzzles via a co-evolutionary process [14].
